# An integrative approach for measuring semantic similarities using gene ontology

**DOI:** 10.1186/1752-0509-8-S5-S8

**Published:** 2014-12-12

**Authors:** Jiajie Peng, Hongxiang Li, Qinghua Jiang, Yadong Wang, Jin Chen

**Affiliations:** 1 School of Computer Science and Technology, Harbin Institute of Technology, Harbin, China; 2 MSU-DOE Plant Research Laboratory, Michigan State University, East Lansing, MI 48824, USA; 3 School of Life Science and Technology, Harbin Institute of Technology, Harbin, China; 4 Department of Computer Science and Engineering, Michigan State University, East Lansing, MI 48824, USA

**Keywords:** Gene ontology, Semantic similarity, Integrative approach

## Abstract

**Background:**

Gene Ontology (GO) provides rich information and a convenient way to study gene functional similarity, which has been successfully used in various applications. However, the existing GO based similarity measurements have limited functions for only a subset of GO information is considered in each measure. An appropriate integration of the existing measures to take into account more information in GO is demanding.

**Results:**

We propose a novel integrative measure called *InteGO*2 to automatically select appropriate seed measures and then to integrate them using a metaheuristic search method. The experiment results show that *InteGO*2 significantly improves the performance of gene similarity in human, Arabidopsis and yeast on both molecular function and biological process GO categories.

**Conclusions:**

*InteGO*2 computes gene-to-gene similarities more accurately than tested existing measures and has high robustness. The supplementary document and software are available at http://mlg.hit.edu.cn:8082/.

## Background

The Gene Ontology (GO) provides a representation of biological knowledge through structured, controlled vocabulary of terms, which are interrelated forming a directed acyclic graph (DAG) for describing the functional information of gene products [1,2]. GO consists of three categories that shared by all organisms: molecular function (MF), biological process (BP) and cellular component (CC) [1]. As a widely used bioinformatics resource, GO provides rich information and a convenient way to study gene functional similarity, which has been successfully used in various aspects including predicting gene functional associations [3], homology analysis [4], assessing target gene functions [5], and predicting subcellular localization [6].

Since GO was released, various computational measurements have been developed to compute gene functional similarities by comparing GO terms with which the genes are annotated [7-23]. These term- comparison measurements can be classified into three categories based on the types of knowledge in GO that they used: edge-based, node-based, and hybrid [18].

The measures in the edge-based category take the structure of GO into account [11,12,22]. By using the topological information of GO directed acyclic graph (DAG), a recently designed method Relative Specificity Similarity (RSS) models both the distance of given term pair to its closest leaf terms and the distance to their most recent common ancestor (MRCA) [22]. The edge-based measures, however, are still fully dependent on the topology of GO DAG, and it is inappropriate to simply equalize the terms at the same topological level [18].

In the node-based category, methods originally designed for natural language processing [24-26] are utilized for term comparisons. In the earlier developed measures, the similarity of two GO terms is defined as the information content of their most informative common ancestor (MICA), indicating its specificity. It was further advanced by modeling the distance between a given term pair to its MICA [13]. The results show strong correlations with yeast gene co-expressions and protein sequence similarities [24,27]. However, the node-based measures only consider the annotations and common ancestors, neglecting the complex topology of the GO DAG.

Hybrid measurements have been recently proposed to consider the more complete information in GO. [15] utilizes all of the parent terms of the target terms, which takes the topology of the GO DAG into account. Hybrid Relative Specificity Similarity (HRSS) employs the concepts of information content, adapting topology, annotations and MICA [22]. The experiment results show that both Wang and HRSS measures perform better than the traditional node-based measures [15,22]. However, these measures still only focus on several types of information in GO but neglect others.

Since none of the existing measure can employ all the information in GO, an integrative approach to unite all the strength of existing measures is preferred. In this direction, [23] proposed a rank-based gene semantic similarity measure called InteGO by synergistically integrating multiple similarity measures (called seed measures) to take into account more aspects of GO (structure, annotation, MICA, MRCA, all of the common parent, etc). InteGO first selects measures based on an evaluation set, and then integrates the selected measures using one of four straightforward methods (maximum, minimum, average and median). The experiment results showed that InteGO performs significant better than the seed measures [23]. However, the performance of InteGO is still limited, because it is vulnerable to the selection of low performance measures, and its fixed integration strategy may not be suitable for all gene pairs.

In this paper, we aimed to present a new integrative measure called *InteGO*2, by choosing the most appropriate seed measures for each gene pair from a pool of candidate measures using a grouping method, and by integrating the selected seed measures using a metaheuristic search method. The major contributions are:

^* ^Our new integrative measure not only takes into account the state-of-the-art GO based measures, but also selects the most appropriate seed measures for each gene pair.

^* ^A metaheuristic search method is presented in *InteGO*2 to flexibly integrate multiple seed measures.

## Method

**Figure 1:**
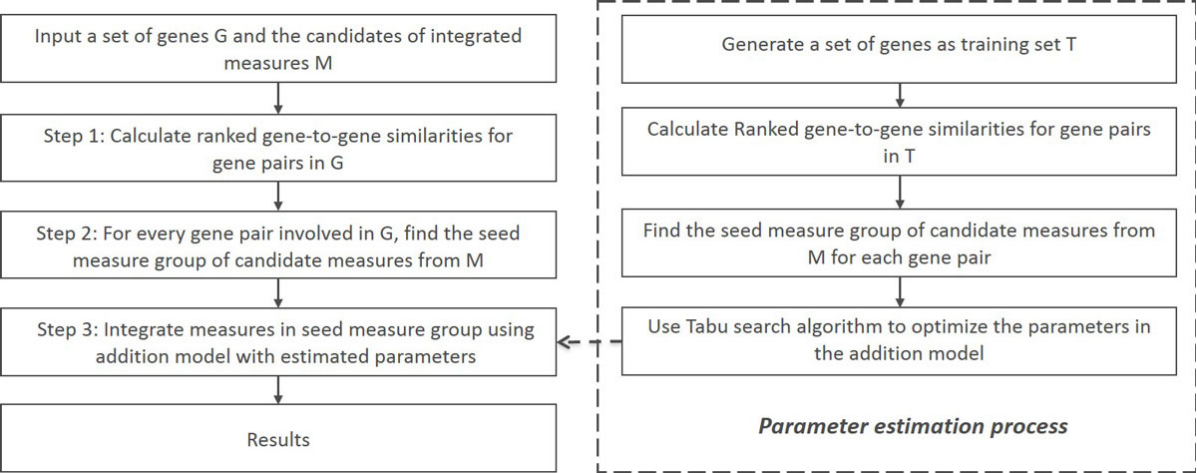
**The Framework of *InteGO*2**. Framework of *InteGO*2 for calculating gene-to-gene similarities for a input gene set (left) and for estimating the parameters in the integration model (right).

The framework of *InteGO*2 is shown in Figure 1. The whole process includes two parts: 1) model training (right), in which the parameters of *InteGO*2 are obtained using a training set *T *, and 2) gene-to-gene similarity calculation (left) for the input gene set *G*. In *InteGO*2, we solve two key problems, i.e, to select the most appropriate seed measures for each gene pair from all the candidate measures and to appropriately integrate the seed measures.

*InteGO*2 has three steps. First, we calculate all the similarity scores using all the candidate measures and then rank them, resulting in a ranked matrix *M_r _*. Second, a grouping process is applied on *M_r _*to identify the common features of all the ranked results, with which we define a set of seed measures for each gene pair saved in *S_seed_*. Third, we integrate all the measures in *S_seed _*with an addition model, in which the parameter of each component is estimated by applying a learning process on training set *T *. We will introduce the three steps of *InteGO*2 in the following text.

### Step 1. Computing similarities using all measures

The similarity scores of all the gene pairs in a given gene set *GS *are calculated using all the candidate measures *S_all_*. And then for each measure, all the gene pairs are sorted incrementally according to their similarity scores, resulting in a ranked matrix *M_r _*, in which each row is a gene pair and each column is a measure, and *M_r _*(*i, j*) is the rank of gene pair *i *in measure *j*. Subsequently, the ranked gene similarity score *RankSim*(*g*_1_*g*_2_*, m*) for genes *g*_1 _and *g*_2 _in *GS *is calculated as:

(1) $$RankSim\left(g_{1}g_{2},m\right)=\frac{2\times M_{r}\left(g_{1}g_{2},m\right)}{{\left|GS\right|}^{2}}$$

where *g*_1 _and *g*_2 _are two target genes, *m *is a candidate measure in *S_all_, |GS| *is the number of genes in gene set *GS*, which according to Figure 1, is the input gene set *G *or the training set *T *. *RankSim*(*g*_1_*g*_2_*, m*) ∈ [0, 1]. *RankSim*(*g*_1_*g*_2_*, m*) indicates how similar *g*_1 _and *g*_2 _is, compared with all of the gene pairs in *GS*. Note that although the similarities using each measure may at a different scale or have a different distribution, the ranked results are comparable. Therefore, the integration of all the ranked results may better reflect functional similarity.

### Step 2. Selecting seed measures

Since different similarity measures use different types of information in GO, or model data in different ways, one measure may perform the best on certain functional categories but not on the others. Alternatively, the integration of suitable measures makes it possible to calculate the overall similarity score by considering all the aspects of GO. A key problem here is to select the most appropriate measures (called seed measures) for every gene pair from a pool of candidate measures.

**Figure 2:**
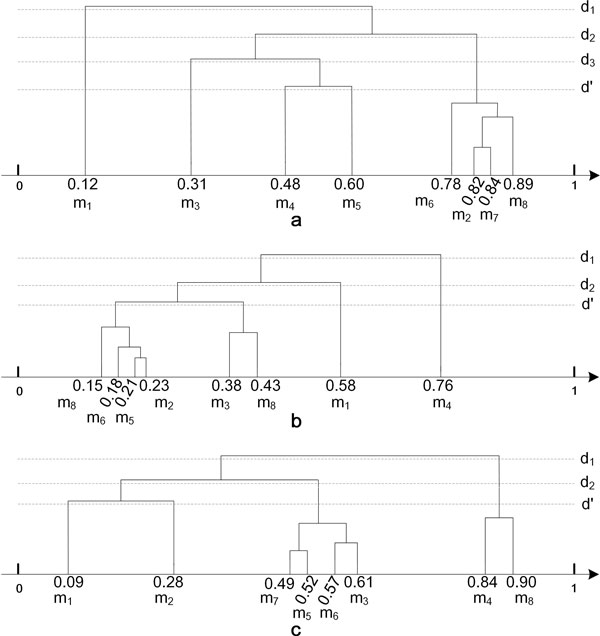
**Illustrative example of three types of seed measure group**. m_1_, m_2_, m_3_,...,m_8 _are eight candidate measures. The values on the number axis are their RankSim values. (a), (b) and (c) are illustration examples of high, low and mix seed measure groups respectively.

In this paper, we present a solution to this problem based on only one principle that *the final ranked score should be the score that all the seed measures agree*. To this end, a grouping algorithm to select the most appropriate seed measures for each gene pair is proposed as follows. Let *RankSim*(*g*_1_*, g*_2_*, m*_1_), *RankSim*(*g*_1_*, g*_2_*, m*_2_), *…, RankSim*(*g*_1_*, g*_2_*, m_n_*) be the ranked similarity scores of *n *candidate measures for *g*_1 _and *g*_2_, and *m_x _*∈ *S_all_*. By putting them on a number axis, we group all the candidate measures agglomeratively based on their distances on the axis, forming a dendrogram *D*(*g*_1_*g*_2_). And then we gradually reduce the distance threshold *d *in *D*(*g*_1_*g*_2_) to iteratively find the isolated measures and remove them until a core group of measures is leftover - which is called the seed measure group (see examples in Figure 2). Mathematically, a seed measure group is the largest group with at least *c *measures, where *c *is a pre-defined value (*c *= 3 in our settings; more detail about the choice of *c *is shown in Additional file 1). And the distance between genes in the seed measure group is not larger than $d^{\prime }$, where $d^{\prime }$ is a pre-defined value ($d^{\prime }=0.10$in our settings; more detail about the choice of $d^{\prime }$ is shown in Additional file 2).For *g*_1_*g*_2_, only the measures in the seed measure group are considered as seed measures, saved in *S_seed_*.

An illustration example of the seed measure group is shown in Figure 2(a). In the figure, with the decrease of *d *from *d*_1 _to $d^{\prime }$, the isolated measures are in the order of *m*_1_, *m*_3_, *m*_4_, and *m*_5_, and the the seed measure group include *m*_2_, *m*_6_, *m*_7_, and *m*_8_.

It is clear that a seed measure group can be labeled as as *high, low*, or *mix *according to its distribution in the number axis. Mathematically, we define the label of a seed measure group using the highest number of the isolated measures in the leftmost, middle or rightmost of the number axis. For example, the seed measure group in Figure 2(a) is high, in Figure 2(b) is low, and in Figure 2(c) is mix. We label the seed measure groups, because the integration strategy could be different for different seed measure group types.

### Step 3. Integrating seed measures

In order to integrate the selected seed measures, we adopt an addition model which is one of the best known method for integrating a number of alternatives [28]. Given a gene pair, we have learned its seed measures and the type of seed measure group from the previous step. For different types of seed measure groups, we build an addition model as shown in Eq. 2:

(2) $$Sim\;\left(g1,g2\right)=\left\{\begin{array}{cc} \sum H_{i}\cdot RankSim\left(i\right)+H\alpha \cdot max+H\beta \cdot min+H\gamma \cdot ave & \text{if type = high;}\\ \sum L_{i}\cdot RankSim\left(i\right)+L\alpha \cdot max+L\beta \cdot min+L\gamma \cdot ave & \text{if type = low;}\\ \sum M_{i}\cdot RankSim\left(i\right)+M\alpha \cdot max+M\beta \cdot min+M\gamma \cdot ave & \text{if type = mix}\text{.} \end{array}\right)$$

where *type *is the type of seed measure group; *i *is a seed measure in the seed measure group; *RankSim*(*i*) is the similarity of given gene pair calculated with measure *i *(Eq. 1); *X_i _*is the parameter of seed measure *i*, where *X *is *H, M *or *L; max, min *and *ave *represent the maximum, minimum and average of all the *RankSim *values for *g*_1 _and *g*_2 _using all the seed measures; and *X_α_, X_β _, X*_γ _are their parameters respectively. We include maximum, minimum and average in the Eq. 2, because the experiment results in [23] show that maximal, minimal and average values are better than individual measure in the tested conditions.

In order to use Eq. 2 for seed measure integration, the parameters, e.g. *X_α_, X_β _, X*_γ _, needs to be assigned. Instead of leaving the difficult job to the end users, we estimate these parameters using a training data *T *. Specifically, we adopt a metaheuristic search method to gradually update the parameters in Eq. 2 to maximize the score of an objective function in *T *.

There are a wide variety of metaheuristics, including simulated annealing, tabu search, iterated local search, variable neighborhood search, and greedy randomized adaptive search. It also includes a learning component to the search, such as ant colony optimization, evolutionary computation, and genetic algorithm. In this paper, we adopt the tabu search method. Comparing with a simple local search procedure, tabu search carefully explores the neighborhood of each solution through the use of memory structures (tabu list) to avoid sticking in the poor-scoring areas or areas where scores plateau [29]. Specifically, given the training set *T *, we use the EC number (Enzyme Commission) to explain molecular function with the criteria that the molecular functions of a group of genes are similar if they have the same EC numbers [15,30,31]. Therefore, we can locate the best candidates of solutions for next move in the searching process.

Given all the genes in *T *grouped by their EC numbers, we compute both the intra-EC gene similarities and the inter-EC gene similarities using Eq. 2 starting with a set of random parameters. We then gradually update the parameters to increase the difference between the intra- and inter-EC similarities. Quantitatively, we utilize the logged fold change (LogFC) measure which has been widely used in the gene expression studies [32]. The LogFC score of EC number *e_i_*is defined in Eq. 3:

(3) $$LogFC\left(e_{i}\right)=\frac{1}{\left|EC\right|}\times \underset{e_{j}\in EC;G\left(e_{j}\right)\cap G\left(e_{i}\right)=\theta }{\sum }\frac{\underset{g\in G\left(e_{i}\right)}{\sum }diff_{g}\left(e_{i},e_{j}\right)}{\left|G\left(e_{j}\right)\right|}$$

where *G*(*e_i_*) is set of all of genes which are assigned to *e_i_; EC *is a set of ECs which do not have any overlapped genes with *e_i _*(*G*(*e_j _*) *∩ G*(*e_i_*) = *∅*) in the training set *T *; and *diff_g _*(*e_i_, e_j _*) is calculated as:

(4) $${diff}_{g}\left(e_{i},e_{j}\right)=\text{ln}\frac{|G\left(e_{i}\right)|\times \underset{g\prime \in G\left(e_{j}\right)}{\sum }\left(1-Sim\;\left(g,g\prime ,t\right)+c\right)}{|G\left(e_{j}\right)|\times \underset{g^{*}\in G\left(e_{i}\right)}{\sum }\left(1-Sim\;\left(g,g^{*},t\right)+c\right)}$$

where *c *is a constant small positive number, as a Laplacian smoothing parameter; *G*(*e_i_*) is the set of all of the genes which EC number is *e_i _*except gene *g; G*(*e_j _*) is the set of all of the genes which EC number is *e_j _; g *is a gene assigned to *e_i_*. *Sim*(*g, g^′^, t*) and *Sim*(*g, g^∗^, t*) are defined in Eq. 2. In Eq. 4, the numerator and denominator represent the inter-EC distance and intra-EC distance respectively. The higher the *diff_g _*(*e_i_, e_j _*) is, the more obvious the positive difference between inter-EC difference and intra-EC difference is.

Finally, given training set *T *grouped by a set of EC numbers, the optimization function for each tabu search move is the average LogFC score of all the involved EC numbers in the training set *T *:

(5) $$OptF\left(T\right)=\frac{1}{\left|T\right|}\times \underset{e_{i}\in T}{\sum }LogFC\left(e_{i}\right)$$

**Figure 3:**
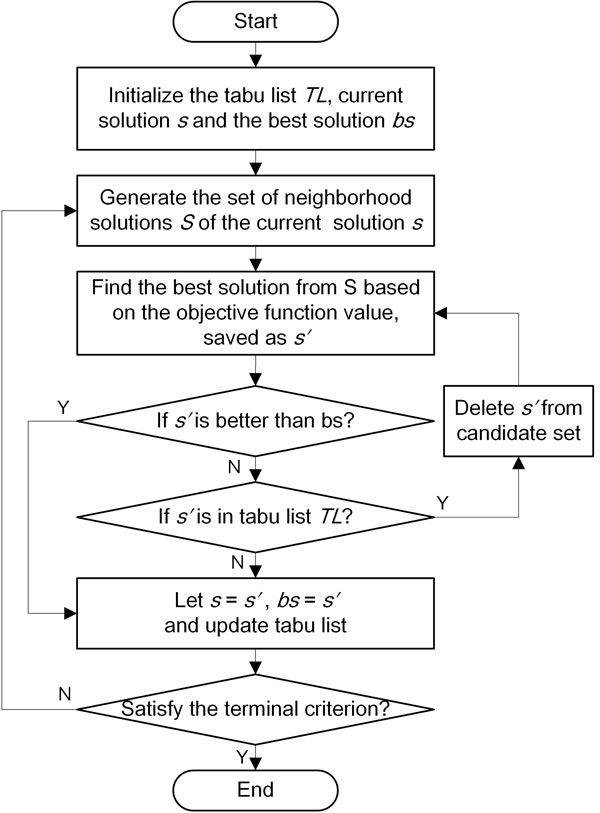
**The flowchart of tabu search process**. The tabu search process is shown step by step in the flowchart.

Subsequently, we estimate the parameters in Eq. 2 using the following tabu search process (Figure 3):

1. Initialize *TL *as the empty tabu list, and a set of random parameters in Eq. 2 as current solution *s *(starting point) satisfying ∑_i∈MG_*X_i _*+ *X_α _*+ *X_β _*+ *X*_γ _= 1.0, where *X *is *H, M *, or *L*. The initial best solution is *bs *= *s*.

2. Calculate the neighborhood solutions of *s *by increasing or decreasing one or multiple parameters in *s*. Note that we learn one group of parameters at a time. For example, while learning parameters for *H_x_*, the other two groups *L_x _*and *M_x _*are fixed.

3. The best solution for next move s′ is selected from the neighborhood solutions of *s *using the optimization function (Eq. 5).

4. If s′ *> bs*, let s′ be the current solution, update *TL *and *bs *= s′.

5. If s′ *≤ bs*, we still let current best solution *s *= s′ and update *TL *if s′ ∉ *TL*. Otherwise, we delete s′ from the neighborhood solutions and go back to step 3.

6. Repeat step 2 to 5 till *bs *is stable.

7. To avoid bias, we repeat step 1 to 6 multiple times and choose the best result.

## Results

We evaluate *InteGO*2 on three model organisms (human, Arabidopsis and Yeast) with different levels of GO annotation scale and complexity [33]. For each of them, we use EC numbers and pathways as independent biological evidences for molecular function and biological process category in GO respectively. Finally, we test the robustness of *InteGO*2 by gradually removing seed measures with best performance.

### Data preparation

The GO annotation and structure data were downloaded from the GO website (http://www.geneontology.org/GO.downloads.shtml). The EC number and pathway information of human, Arabidopsis and Yeast were downloaded from the HumanCyc (http://humancyc.org), PlantCyc (http://ftp.plantcyc.org/Pathways) and Saccharomyces genome database (http://www.yeastgenome.org/download-data/curation) respectively. *InteGO*2 was implemented with Python 2.7 with NetworkX package (http://networkx.github.io).

### Performance evaluation on molecular function

Proteins sharing the same EC numbers are considered to have similar molecular functions. For every manually curated pathway in human, Arabidopsis and yeast, we grouped the genes based on their EC numbers (full four digits) and tested the difference between the inter- and intra-group gene-gene similarities. There are in total 125, 205 and 32 EC groups with least three genes in human, Arabidopsis and yeast respectively.

In the experiments, we chose seven widely used measures in all the three categories as candidate measures. We also added a fake measure to simulate the situation where a wrong measure was included to test the robustness of *InteGO*2. Among the seven measures, SimUI [34] and TO [35] measure use the GO annotations information directly; Resnik [24], Schlicker [13] and SimGIC [36] measure use annotation information to calculate the information content of GO terms; Wang [15] measure considers the complex topology of GO; HRSS [22] considers the shared path based on information content. More detail description is shown in Additional file 3. In the fake measure, a random half of the similarity scores were computed with Resnik measure, and the other half were 1 or 0, such that the similarity of two genes with the same EC is 0, otherwise it is 1 (the reversed values ensure that the fake measure has low quality).

In order to evaluate *InteGO*2 systematically, we adopted the cross-validation strategy by randomly selecting 1*/*5 of human ECs as the testing set (200 genes involved) and the other 4*/*5 of human ECs being the training set (823 genes involved). The same training set was used for Arabidopsis and yeast (1151 and 121 genes involved respectively). Using the training set, the parameters in Eq. 2 were estimated, which were directly applied on the testing set to compute the EC-based LogFC scores using Eq. 5.

We found that the parameters for the three types of seed measure groups (high, low and mix) are significantly different, reflecting different integration strategies. The highest parameter in the high seed measure groups is maximum, in the low seed measure groups is minimum, and in the mix seed measure groups is simUI measure.

**Figure 4:**
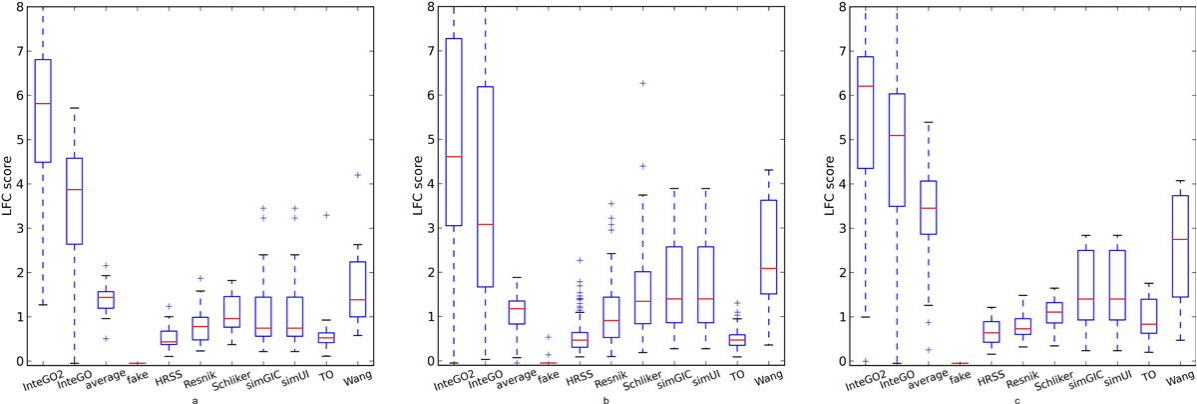
**LogFC score comparison in Molecular Function category on human (a), Arabidopsis (b) and yeast (c)**. LogFC score comparison for eight candidate measures (fake, HRSS, Resnik, Schlicker, simGIC, simUI, TO and Wang) and three integration measures average, InteGO and *InteGO*2 in Molecular Function category on human (a), Arabidopsis (b) and yeast (c). The top and bottom of the boxes represent 75th and 25th percentiles, red lines are the median, top and bottom whiskers represent greatest and lowest values except outliers. Cross nodes represent outliers that are larger than the sum of 75th and 1.5 interquartile range.

**Figure 5:**
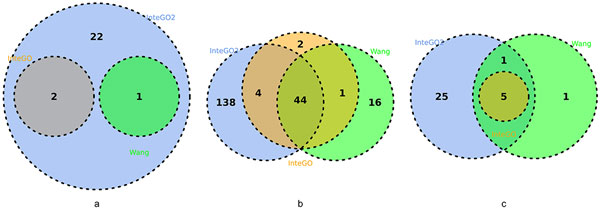
**Venn Diagram for *InteGO*2, InteGO and Wang in Molecular Function category on human (a), Arabidopsis (b) and yeast (c)**. Venn Diagram for *InteGO*2, InteGO and Wang measure with number of ECs on which perform best on human (a), Arabidopsis (b) and yeast (c).

We compared the performance of *InteGO*2 with all the candidate measures, the average value of them and InteGO. Figure 4 shows that *InteGO*2 performed the best among all the measures in all the three species. For example, the median, 75th and 25th percentile of LogFC scores of *InteGO*2 on human were 5.9, 6.9 and 4.5, significantly higher than the seed measures it integrated (Figure 4(a) and supplementary table S1 in Additional file 4). Interestingly, the performance of *InteGO*2 was significantly higher than our previous measure InteGO, indicating that adding a weak measure has almost negligible effect to *InteGO*2, but can significantly affect InteGO. Comparing the LogFC scores on every EC group using *InteGO*2, InteGO and Wang measure (the best seed measure), we found that *InteGO*2 performed the best in all 25 ECs in the testing set, while InteGO and Wang measure were being the best in 2 or 1 ECs only (Figure 5(a)). Similarly, the median of LogFC scores of *InteGO*2 in Arabidopsis is 4.6, which is 1.5-fold higher than InteGO (Figure 4(b) and supplementary table S2 in Additional file 4). *InteGO*2 performed the best in 186 of 205 ECs, while Wang performed the best in 61 ECs (Figure 5(b)). We also evaluated *InteGO*2 on yeast which has richer information in GO than human and Arabidopsis. *InteGO*2 performed the best with the median LogFC score being 6.2 (Figure 4(c) and supplementary table S3 in Additional file 4). it was the best in 31 out of 32 total EC groups (Figure 5(c)).

Statistics analysis was carried out to test the significance of *InteGO2 *results. The p-values of t-test indicate that the results of *InteGO2 *are significantly different with the results of other measures except simGIC, simUI and Wang measure on Arabidopsis and yeast (T-Test, supplementary Table S4 in Additional file 4).

### Performance evaluation on biological process

Given that genes annotated to the similar biological process may be involved in the same manually curated pathway, we grouped genes based on the pathway information, and on these gene groups we evaluated *InteGO*2. There are in total 258, 154 and 141 pathways with at least two genes in humanCyc, PlantCyc and Saccharomyces genome database respectively.

**Figure 6:**
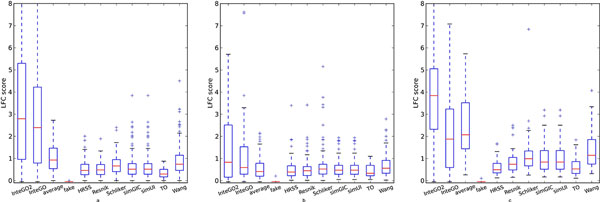
**LogFC score comparison in Biological Process category on human (a), Arabidopsis (b) and yeast (c)**. LogFC score comparison for eight candidate measures (fake, HRSS, Resnik, Schlicker, simGIC, simUI, TO and Wang) and three integration measures average, InteGO and InteGO2 in Biological Process (BP) category on human(a), Arabidopsis(b) and yeast(c). The top and bottom of the boxes represent 75th and 25th percentiles, red lines are the median, top and bottom whiskers represent greatest and lowest values except outliers. Cross nodes represent outliers that are larger than the sum of 75th and 1.5 interquartile range.

**Figure 7:**
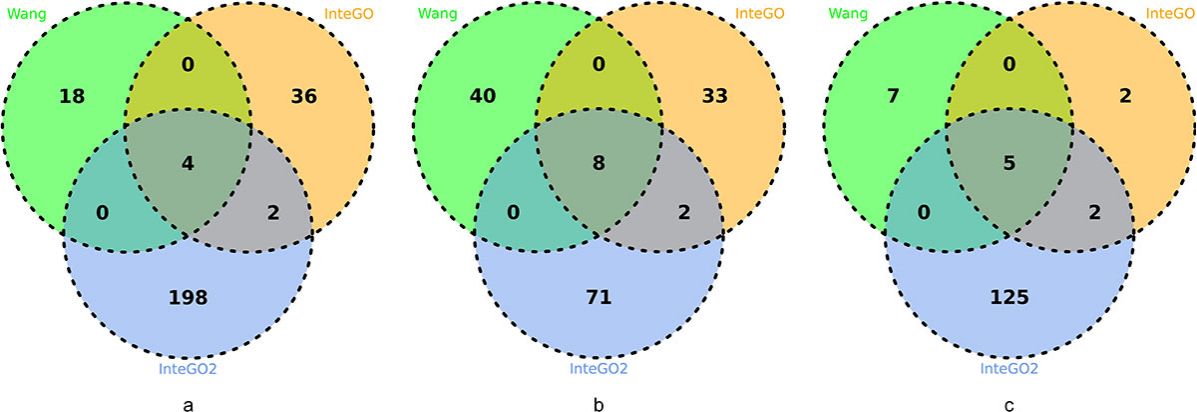
**Venn Diagram for *InteGO*2, InteGO and Wang in Biological Process category on human (a), Arabidopsis (b) and yeast (c)**. Venn Diagram for *InteGO*2, InteGO and Wang measure with number of Pathways on which perform best on human(a), Arabidopsis(b) and yeast(c).

The same LogFC method (Eq. 3) were used in the performance test. In human and Arabidopsis, the median and 75th percentile of LogFC scores of LogFC scores were higher than other measures (Figure 6(a), (b) and supplementary table S5 and S6 in Additional file 4), indicating that integrating multiple gene similarity measures with *InteGO*2 could increase the overall performance. Comparing the LogFC scores from the *InteGO*2, InteGO and Wang measure for each pathway, Figure 7(a) and (b) show that *InteGO*2 performs best in 204 of 258 pathways and 81 of 154 pathways on human and Arabidopsis respectively. In yeast, the performance of *InteGO*2 is still the best. The median, 75th percentile and 25th percentile of LogFC scores are 3.9, 5.0 and 2.3, which are significant higher than the second-best measure InteGO (Figure 6(c) and supplementary table S7 in Additional file 4). In addition, *InteGO*2 performs best in 132 of 141 (93.6%) yeast pathways (Figure 7(c)). Although *InteGO*2 perform well in most datasets, its performance on Arabidopsis is not good enough (the median of LogFC score is around 1). The reason may be that all the result of seed measures are not good and very close to each other. Therefore, the grouping process (see subsection 2.2) in *InteGO*2 cannot select the appropriate seed measures from the seed measure. Even though, *InteGO*2 also increase the performance of the similarity measures.

Statistics analysis was carried out to test the significance of *InteGO2 *results. The p-values of t-test indicate that the results of *InteGO2 *are significantly different with the results of other measures except simGIC, simUI and Wang measure on Arabidopsis (T-Test, supplementary Table S8 in Additional file 4).

The results indicate that *InteGO*2 successfully utilizes the GO information by integrating seed measures appropriately to better deliver functional similarities better genes.

### Robustness of *InteGO*2

**Figure 8:**
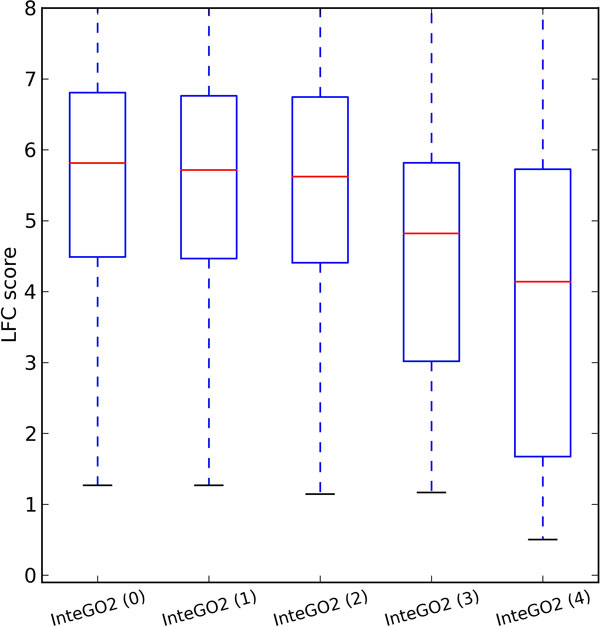
**The Robustness test of *InteGO*2**. The Robustness test of *InteGO*2 on molecular function based on human EC.

To test the robustness of *InteGO*2, we gradually removed a candidate measure (Wang, Schlicker, Resnik, simUI) and then compute the logFC score. Figure 8 shows that the performance reduced slowly by removing the first two measures (supplementary table S9 in Additional file 4). The median of LogFC decreased less than 1.0 after removing three best measures. This is because *InteGO*2 can select the most appropriate seed measures for each gene pair, since no measurement is suitable for every gene pair. To analysis the contribution of the different measures to the overall similarity, we applied leave-one-out measure on InteGO2. The result shows that InteGO2 is overall robust to remove any integrated measure (Additional file 5). The performance of InteGO2 decreases most after Resnik measure is removed.

### Performance evaluation on protein sequences

**Figure 9:**
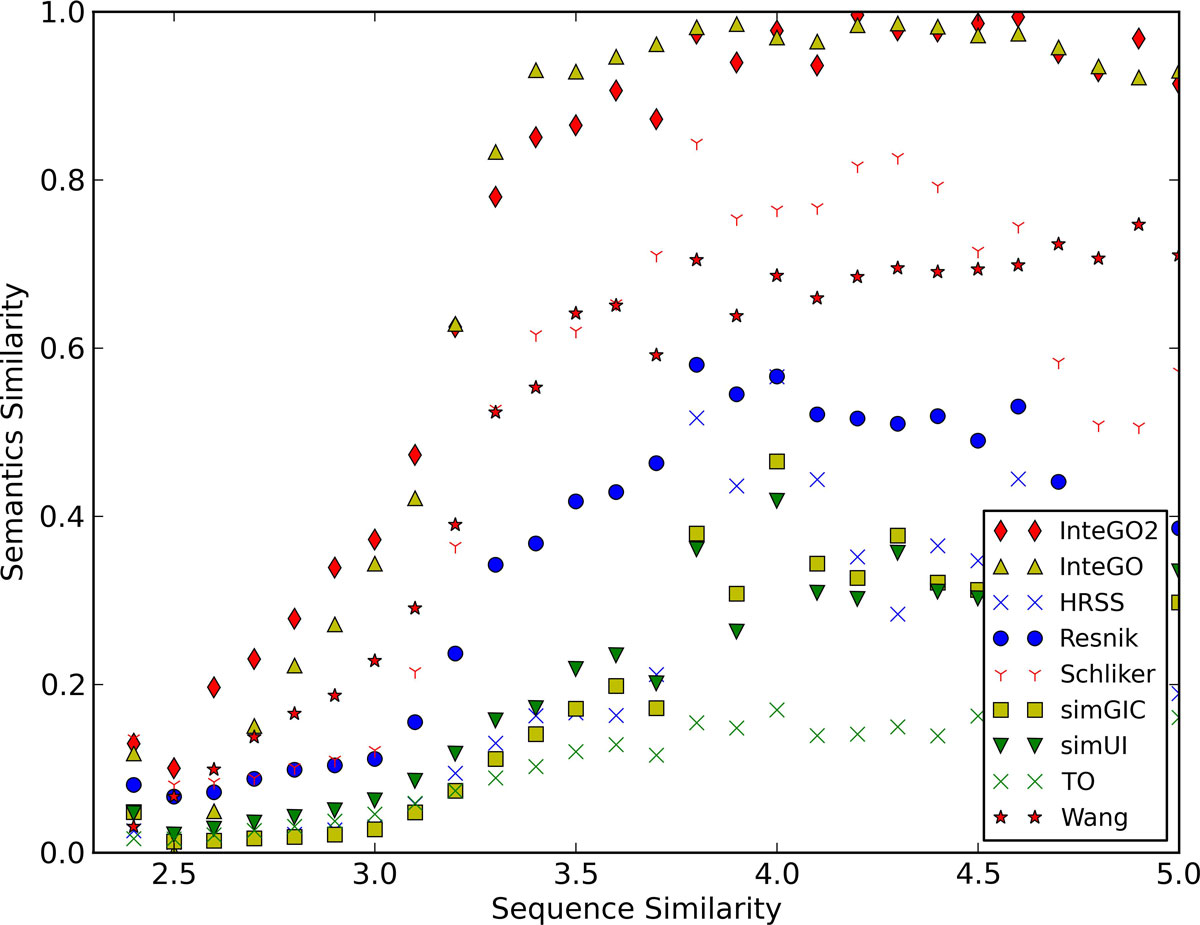
**Comparing InteGO2 with other measures with protein sequence similarity on on human**. The x-axis is the BLAST sequence similarity and y-axis is the normalized semantic similarity based on GO.

In addition to use the logFC score as the evaluation criteria, we used protein sequence similarity as an independent evidence for further performance evaluation on the molecular function category [18]. In this experiment, the same human gene set in subsection "Performance evaluation on molecular function" was used, and the sequence similarity scores (*ln*(*BitScore*)) were calculated with BLAST [37]. Figure 9 shows that among all the GO based semantic similarity measures, *InteGO*2 has the highest correlation score with the sequence based similarity with R-Squared 0.96 (polynomial model; Supplementary Table S10 in Additional file 4).

### Generating functional association maps

**Figure 10:**
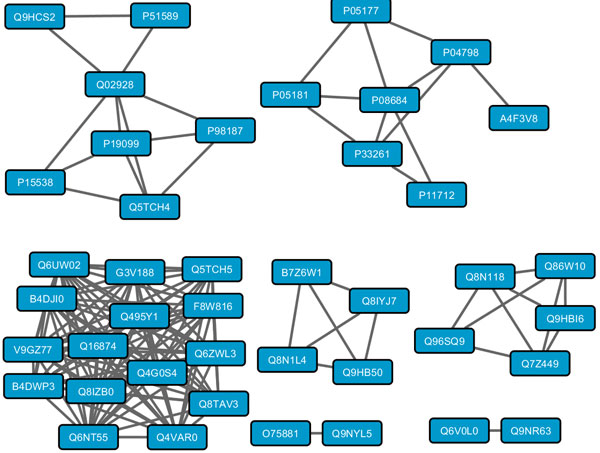
**The human P540 gene functional association map**. The human P540 gene functional association map with 42 genes and 145 edges.

Since *InteGO*2 computes gene-to-gene similarities more accurately than the tested existing measures, we computed the gene similarity scores for all the human, Arabidopsis and yeast genes on both molecular function and biological process GO categories, and generated a functional association map for each organism. As a demonstration, the human P540 [38] gene functional association map (*Sim*(*g*1*g*2) = 1.0) with 42 genes and 145 edges consists a tightly connected subgraph and several small or large but sparsely connected subgraphs (see Figure 10). These networks provide a new platform for more advanced biomedical researches which could be beneficial in medical diagnostics.

## Conclusions

The calculation of GO-based gene functional similarity has already been widely applied [3-6]. However, since the existing measurements only use a subset of the GO information (e.g., topology of DAG, annotations, MICA, edge length and all the parents term), the demand to integrate these measurements is compelling.

In this paper, we proposed a new integrative measure called *InteGO*2 by automatically selecting the most appropriate seed measures and by integrating the seed measures using an addition model. First, we calculate the ranked similarity scores using all the measures. Second, seed measures are selected using a grouping process. Third, the parameters of the addition model are estimated by optimizing an objective function on a training data. Experimental results using ECs and pathways show that *InteGO*2 performs the best among all the measures. It also shows that *InteGO*2 is robust against the unavailability of candidate measures. Note that we have proposed InteGO in the previous work to unify different measures [23], which can be considered as a simplified case of *InteGO*2.

To demonstrate the advantages of *InteGO*2, we computed the gene similarity scores for all the human, Arabidopsis and yeast genes on both molecular function and biological process GO categories, and generated a functional association map for each organism. The new functional association maps, together with the existing biological networks, can be beneficial in medical diagnostics, and they also may provide more biological insights into gene function and regulation. In the future, we will apply *InteGO2 *to more organisms, data sets (such as protein-family-based index) and compare the new functional association maps with the existing biological network (such as protein-protein network and genetic interaction network) to predict protein or genetic interaction based on the GO similarity scores.

## Competing interests

The authors declare that they have no competing interests.

## Authors' contributions

**JC **and **YW **designed the algorithm and experiments. **JP **and **HL **implemented the algorithm and finished the experiments. **QJ **helped to design the algorithm to find the seed measure group.

## Supplementary Material

Additional file 1 The effect of varying the least size of the seed measure group on *InteGO2 *performance. The x-axis is the least size of the seed measure group. The y-axis is the LogFC scores. The top and bottom of the boxes represent 75th and 25th percentiles, red lines are the median, top and bottom whiskers represent greatest and lowest values except outliers. Cross nodes represent outliers that are larger than the sum of 75th and 1.5 interquartile range. Click here for file

Additional file 2 The effect of varying the threshold of the distance between genes in the seed measure group on *InteGO2 *performance. The x-axis is the threshold of the distance between genes in the seed measure group. The y-axis is the LogFC scores. The top and bottom of the boxes represent 75th and 25th percentiles, red lines are the median, top and bottom whiskers represent greatest and lowest values except outliers. Cross nodes represent outliers that are larger than the sum of 75th and 1.5 interquartile range. Click here for file

Additional file 3 The description of the integrated measures. Seven individual measures are described in this file. The reference papers of these measures are also listed. Click here for file

Additional file 4 Supplementary tables. All the supplementary tables (ten tables in total) are included in this file. Click here for file

Additional file 5 The effect of removing single integrated measure on *InteGO2 *performance. The x-axis is the individual measure removed. The y-axis is the LogFC scores. The top and bottom of the boxes represent 75th and 25th percentiles, red lines are the median, top and bottom whiskers represent greatest and lowest values except outliers. Cross nodes represent outliers that are larger than the sum of 75th and 1.5 interquartile range. Click here for file

## Declarations

The publication costs for this article were funded by the corresponding author's institution.

This article has been published as part of *BMC systems Biology *Volume 8 Supplement 5, 2014: Proceedings of the 25th International Conference on Genome Informatics (GIW/ISCB-Asia): Systems Biology. The full contents of the supplement are available online at http://www.biomedcentral.com/bmcsystbiol/supplements/8/S5.
