# Supplementary figures and images for: An integrative approach for measuring semantic similarities using gene ontology
Source: BMC Syst Biol. 2014 Dec 12;8(Suppl 5):S8. doi: 10.1186/1752-0509-8-S5-S8 (PMC4305987; doi:10.1186/1752-0509-8-S5-S8)

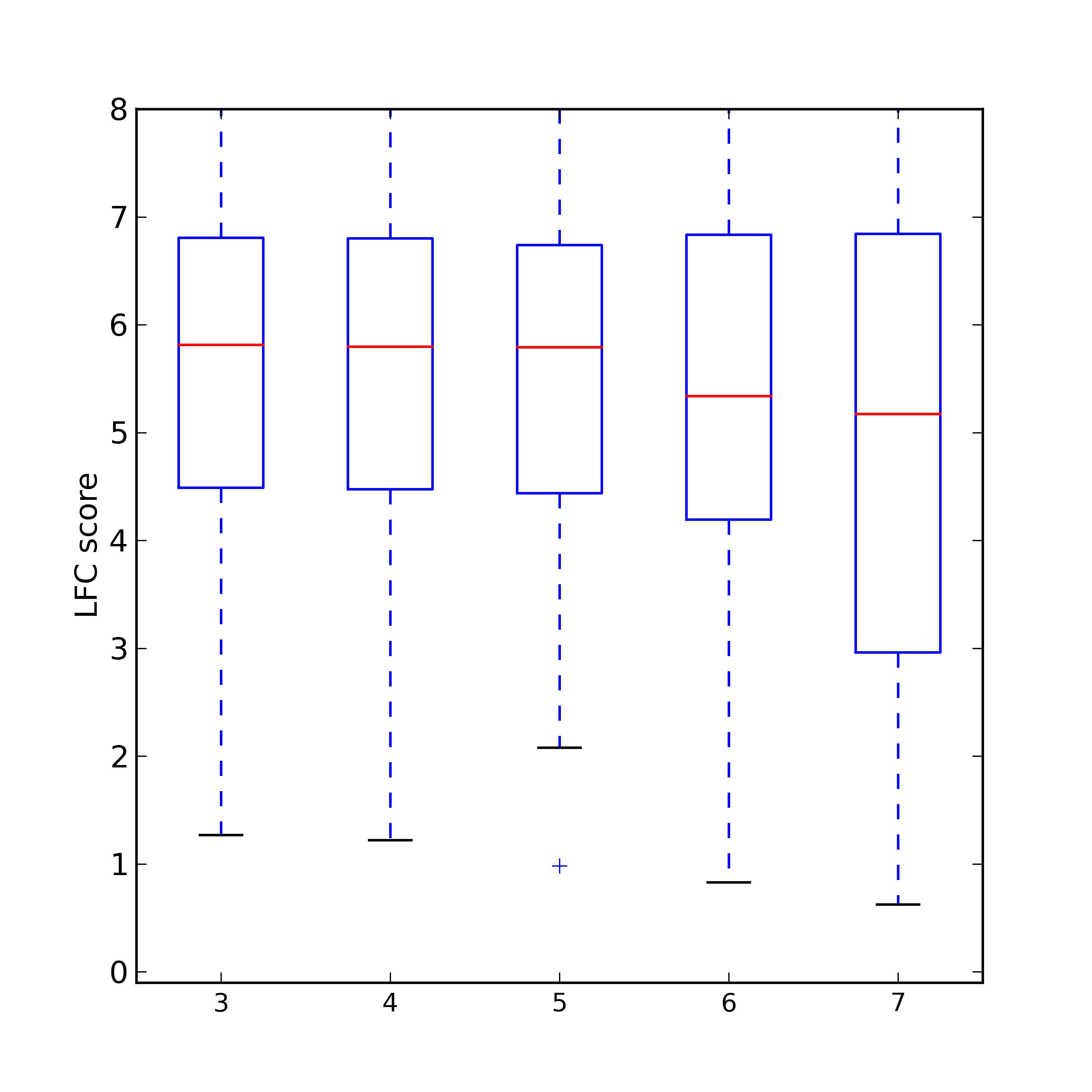

Supplement: Additional file 1 — The effect of varying the least size of the seed measure group on InteGO2 performance. The x-axis is the least size of the seed measure group. The y-axis is the LogFC scores. The top and bottom of the boxes represent 75th and 25th percentiles, red lines are the median, top and bottom whiskers represent greatest and lowest values except outliers. Cross nodes represent outliers that are larger than the sum of 75th and 1.5 interquartile range. [file 1752-0509-8-S5-S8-S1.jpg]

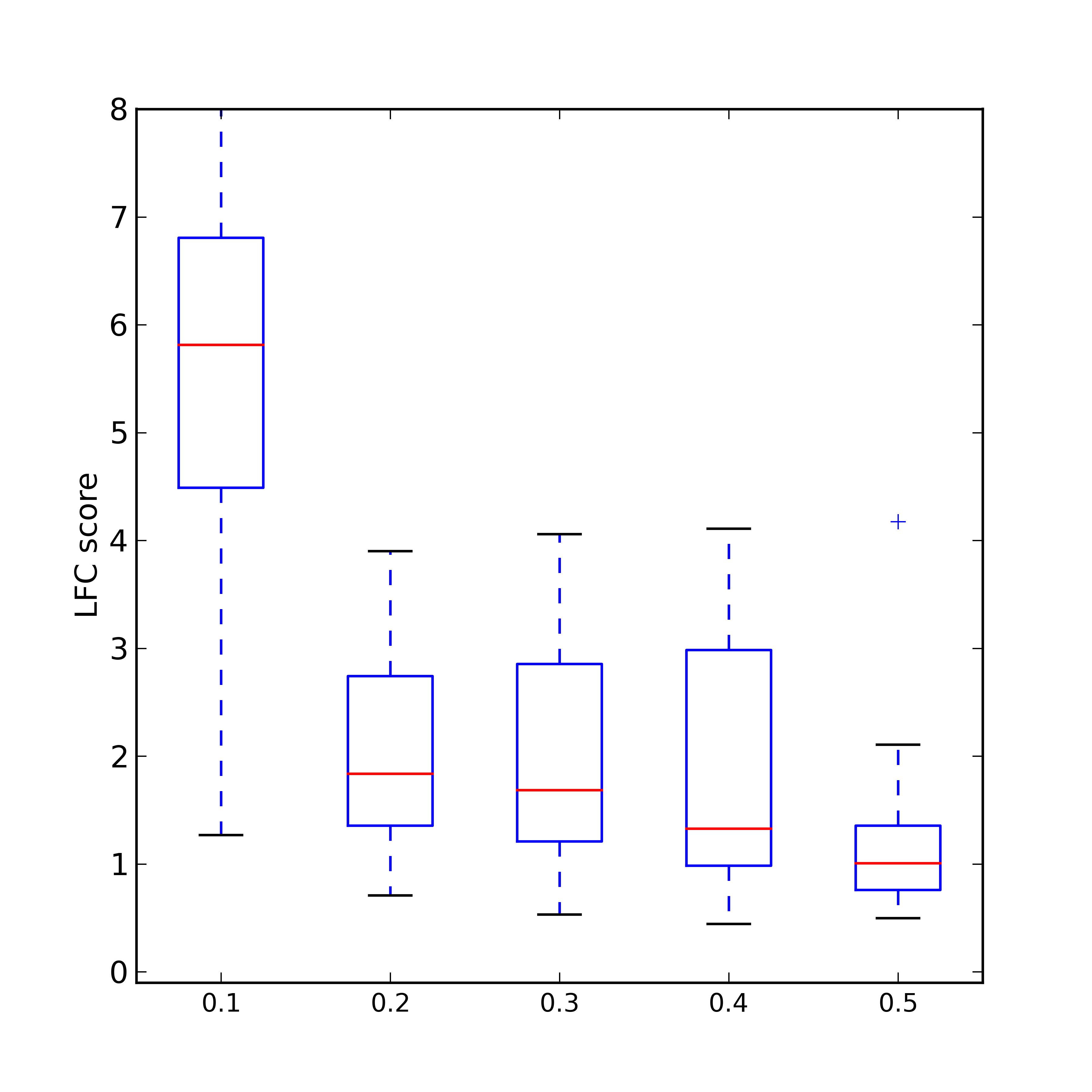

Supplement: Additional file 2 — The effect of varying the threshold of the distance between genes in the seed measure group on InteGO2 performance. The x-axis is the threshold of the distance between genes in the seed measure group. The y-axis is the LogFC scores. The top and bottom of the boxes represent 75th and 25th percentiles, red lines are the median, top and bottom whiskers represent greatest and lowest values except outliers. Cross nodes represent outliers that are larger than the sum of 75th and 1.5 interquartile range. [file 1752-0509-8-S5-S8-S2.jpg]

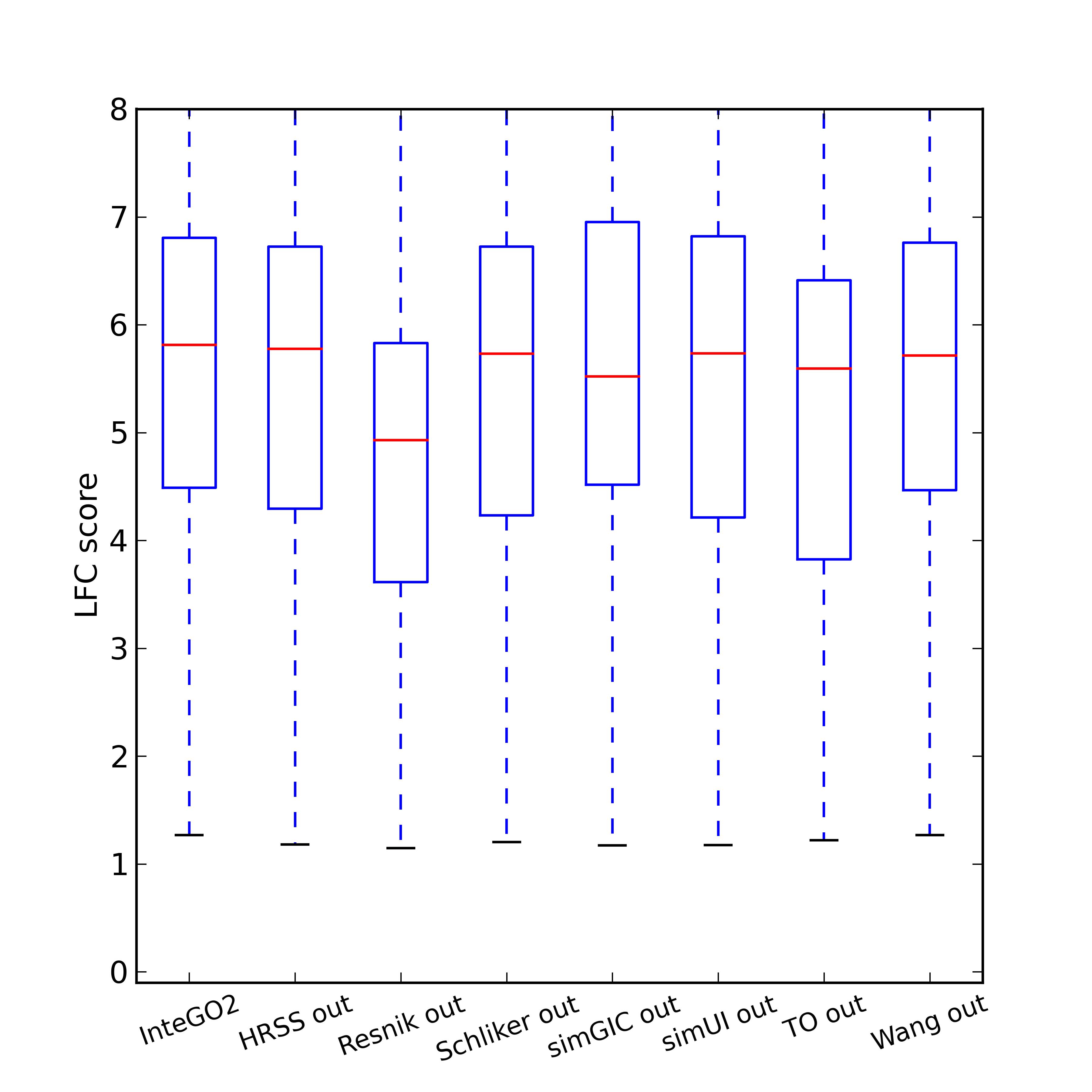

Supplement: Additional file 5 — The effect of removing single integrated measure on InteGO2 performance. The x-axis is the individual measure removed. The y-axis is the LogFC scores. The top and bottom of the boxes represent 75th and 25th percentiles, red lines are the median, top and bottom whiskers represent greatest and lowest values except outliers. Cross nodes represent outliers that are larger than the sum of 75th and 1.5 interquartile range. [file 1752-0509-8-S5-S8-S5.jpg]
