# Supplementary material for: An integrative approach for measuring semantic similarities using gene ontology
Source: BMC Syst Biol. 2014 Dec 12;8(Suppl 5):S8. doi: 10.1186/1752-0509-8-S5-S8 (PMC4305987; doi:10.1186/1752-0509-8-S5-S8)
Supplement: Additional file 3 — The description of the integrated measures. Seven individual measures are described in this file. The reference papers of these measures are also listed. [file 1752-0509-8-S5-S8-S3.pdf]

Introduction to the seven integrated measures

1) Resnik

By combining Information Content (IC) with the ontology structure, Resnik defined a taxonomic similarity as the IC of the lowest common ancestor (LCA) [6], which is then widely used as a similarity measure for GO terms. Let t be a GO term, the information content of t is defined as $IC(t) = -log(|G_t|/|G|)$, where G_t and G are sets of genes annotated to t and the root term (and all its descendants). Let t_a and t_b be two GO terms in the same category and G_{LCA} be the set of gene products annotated to LCA of t_a and t_b, the similarity between t_a and t_b is defined as the information content of LCA:

$$\mathrm{Sim}_{\mathrm{Resnik}}\left(t_a, t_b\right) = IC\left(LCA\right) = -log\frac{|G_{LCA}|}{|G|}$$

Reference: Resnik P: Semantic similarity in a taxonomy: An information-based measure and its application to problems of ambiguity in natural language. Journal of Artificial Intelligence Research 1999, 11:95-130.

2) Schlicker

Schlicker *et al*. normalized the Resnik measure based on the information content of t_a and t_b, and adjusted the overall score with a weighting function:

$$\mathrm{Sim}_{\mathrm{Schlicker}}\left(t_a, t_b\right) = \frac{2 \times IC(LCA)}{IC\left(t_a\right) + IC(t_b)} \times (1 - \frac{|G_{LCA}|}{|G|})$$

Reference: Schlicker A, Domingues F, Rahnenfhrer J, Lengauer T: A new measure for functional similarity of gene products based on Gene Ontology. BMC bioinformatics 2006, 7:302.

3) Wang

Wang *et al*. proposed a measure that considers the topology of the GO graph by taking into account all of the parent terms (instead of just the LCA), but not the gene annotations. Given a term t_a and its parent term p in the GO, the semantic contribution of p to t_a, denoted as $S_{ta,p}$, is defined as the maximal semantic contribution of the paths from t_a to p. The GO term similarity is defined as

$$\mathrm{Sim}_{\mathrm{Wang}}(t_a, t_b) = \frac{\sum_{p \in P_a \cap P_b} (S_{t_{a,p}} + S_{t_{b,p}})}{\sum_{t \in P_a} S_{t_{a,p}} + \sum_{t \in P_b} S_{t_{b,p}}}$$

where P_a (or P_b) are the sets of all the parents of t_a (or t_b).

Reference: Wang J, Du Z, Payattakool R, Philip S, Chen C: A new method to measure the semantic similarity of GO terms. Bioinformatics 2007, 23:1274-1281.

4) HRSS

By using the topological information of GO directed acyclic graph (DAG), Relative Specificity Similarity (RSS) models both the distance of given term pair to its closest leaf terms and the distance to their most recent common ancestor (MRCA). Hybrid Relative Specificity Similarity (HRSS) employs the concepts of information content, adapting topology, annotations and most informative common ancestor (MICA). Given two terms t_1 and t_2, the similarity score between them is calculated based on following equations.

$$\alpha_{IC} = dist_{IC}(root, MICA) = IC(MICA)$$

$$\beta_{IC} = \frac{dist_{IC}(t_1, MIL_1) + dist_{IC}(t_2, MIL_2)}{2}$$

$$\gamma_{IC} = dist(MICA, t_1) + dist(MICA, t_2)$$

$$dist_{IC}(u, v) = IC(v) - IC(u)$$

$$TermSim_{HRSS}(t_1, t_2) = \frac{1}{1+\gamma} \times \frac{\alpha_{IC}}{\alpha_{IC} + \beta_{IC}}$$

where root is the root term of GO category; MICA is the most informative common ancestor of t_1 and t_2; MILi is the most informative child leaf of t_i; dist() represent the length between two terms in GO; IC() is the same as the information content defined in Resnik measure.

Reference: Wu X, Pang E, Lin K, Pei Z: Improving the measurement of semantic similarity between gene ontology terms and gene products: Insights from an edge-and ic-based hybrid method. PloS one 2013, 8:e66745.

5) TO

Given two genes g_1 and g_2, T1 and T2 are the GO terms annotating g1 and g2 respectively. simUI calculates similarity as:

$$GeneSim(g_1, g_2) = | T_1 \cap T_2 |$$

Reference: Lee H, Hsu A, Sajdak J, Qin J, Pavlidis P: Coexpression analysis of human genes across many microarray data sets. Genome research 2004, 14:1085-1094.

6) simUI

Given two genes g_1 and g_2, T1 and T2 are the GO terms annotating g1 and g2 respectively. simUI calculates similarity as:

$$GeneSim(g_1, g_2) = | T_1 \cap T_2 | / | T_1 \cup T_2 |$$

Reference: Gentleman R: Visualizing and distances using GO. URL http://www.bioconductor.org/docs/vignettes.html.

7) simGIC

simGIC is an expansion of simUIwhere instead of counting the terms we sum their information content (IC), which is defined in resnik measure.

$$GeneSim(g_1, g_2) = \frac{\sum_{t \in T_1 \cap T_2} IC(t)}{\sum_{t \in T_1 \cup T_2} IC(t)}$$

Reference: Pesquita C, Faria D, Bastos H, Falcao A, Couto F: Evaluating GO-based semantic similarity measures. In Annual Bio-Ontologies Meeting 2007, :37{40.
