# Supplementary material for: An integrative approach for measuring semantic similarities using gene ontology
Source: BMC Syst Biol. 2014 Dec 12;8(Suppl 5):S8. doi: 10.1186/1752-0509-8-S5-S8 (PMC4305987; doi:10.1186/1752-0509-8-S5-S8)
Supplement: Additional file 4 — Supplementary tables. All the supplementary tables (ten tables in total) are included in this file. [file 1752-0509-8-S5-S8-S4.pdf]

Supplementary Tables of "An Integrative Approach for Measuring Semantic Similarities using Gene Ontology"

Jiajie Peng, Hongxiang Li, Yadong Wang, Jin Chen

August 24, 2014

Table S1: The 25th percentile, median and 75th percentile value of LFC score boxplot on molecular function based on human EC for InteGO2, InteGO, average and 8 seed measures.

	25th percentile	median	75th percentile
InteGO2	4.489	5.929	6.943
InteGO	2.638	3.888	4.986
average	1.192	1.443	1.746
fake	-0.05	-0.05	-0.05
HRSS	0.372	0.46	0.685
Resnik	0.477	0.803	1.029
Schliker	0.764	1.015	1.498
simGIC	0.56	0.825	1.461
simUI	0.56	0.825	1.461
TO	0.415	0.551	0.67
Wang	0.998	1.675	2.396

Table S2: The 25th percentile, median and 75th percentile value of LFC score boxplot on molecular function based on arabidopsis EC for InteGO2, InteGO, average and 8 seed measures.

	25th percentile	median	75th percentile
InteGO2	3.051	4.63	7.861
InteGO	1.668	3.119	7.819
average	0.832	1.176	1.351
fake	-0.05	-0.05	-0.05
HRSS	0.307	0.469	0.641
Resnik	0.526	0.91	1.46
Schliker	0.838	1.346	2.037
simGIC	0.86	1.45	2.625
simUI	0.86	1.45	2.625
TO	0.347	0.469	0.587
Wang	1.509	2.093	3.677

Table S3: The 25th percentile, median and 75th percentile value of LFC score boxplot on molecular function based on yeast EC for InteGO2, InteGO, average and 8 seed measures.

	25th percentile	median	75th percentile
InteGO2	4.465	6.227	6.923
InteGO	3.55	5.091	6.288
average	2.878	3.503	4.121
fake	-0.05	-0.05	-0.05
HRSS	0.424	0.659	0.908
Resnik	0.603	0.734	0.974
Schliker	0.862	1.108	1.351
simGIC	0.968	1.503	2.656
simUI	0.968	1.503	2.656
TO	0.633	0.831	1.394
Wang	1.453	2.761	3.734

Table S4: The P-values for comparing InteGO2 with other measures using t-test on EC.

Measures	human	arabidopsis	yeast
average vs. InteGO2	$<$ 1.0e-07	$<$ 1.0e-07	$<$ 1.0e-07
InteGO vs. InteGO2	$<$ 1.0e-07	$<$ 1.0e-07	$<$ 1.0e-07
fake vs. InteGO2	$<$ 1.0e-07	$<$ 1.0e-07	$<$ 1.0e-07
HRSS vs. InteGO2	$<$ 1.0e-07	$<$ 1.0e-07	$<$ 1.0e-07
Resnik vs. InteGO2	$<$ 1.0e-07	$<$ 1.0e-07	$<$ 1.0e-07
Schliker vs. InteGO2	$<$ 1.0e-07	$<$ 1.0e-07	$<$ 1.0e-07
simGIC vs. InteGO2	$<$ 1.0e-07	8.8e-01	4.6e-01
simUI vs. InteGO2	$<$ 1.0e-07	8.8e-01	4.6e-01
TO vs. InteGO2	$<$ 1.0e-07	$<$ 1.0e-07	$<$ 1.0e-07
Wang vs. InteGO2	$<$ 1.0e-07	2.0e-01	8.0e-01

Table S5: The 25th percentile, median and 75th percentile value of LFC score boxplot on biological progress based on human pathway for InteGO2, InteGO, average and 8 seed measures.

	25th percentile	median	75th percentile
InteGO2	0.954	2.8	5.34
InteGO	0.791	2.42	4.227
average	0.537	0.927	1.473
fake	-0.05	-0.05	-0.05
HRSS	0.267	0.461	0.734
Resnik	0.271	0.477	0.733
Schliker	0.405	0.661	0.953
simGIC	0.285	0.521	0.77
simUI	0.285	0.521	0.77
TO	0.163	0.296	0.508
Wang	0.458	0.739	1.155

Table S6: The 25th percentile, median and 75th percentile value of LFC score boxplot on biological progress based on arabidopsis pathway for InteGO2, InteGO, average and 8 seed measures.

	25th percentile	median	75th percentile
InteGO2	0.15	0.866	2.895
InteGO	0.286	0.597	1.548
average	0.199	0.417	0.788
fake	-0.05	-0.05	-0.05
HRSS	0.221	0.388	0.671
Resnik	0.231	0.447	0.642
Schliker	0.296	0.529	0.746
simGIC	0.285	0.479	0.692
simUI	0.285	0.479	0.692
TO	0.219	0.338	0.688
Wang	0.333	0.563	0.937

Table S7: The 25th percentile, median and 75th percentile value of LFC score boxplot on biological progress based on yeast pathway for InteGO2, InteGO, average and 8 seed measures.

	25th percentile	median	75th percentile
InteGO2	2.316	3.852	5.066
InteGO	0.59	1.967	3.24
average	1.435	2.156	3.533
fake	-0.05	-0.05	-0.05
HRSS	0.347	0.493	0.784
Resnik	0.481	0.739	1.047
Schliker	0.663	0.993	1.386
simGIC	0.515	0.843	1.378
simUI	0.515	0.843	1.378
TO	0.324	0.534	0.862
Wang	0.751	1.153	1.872

Table S8: The P-values for comparing InteGO2 with other measures using t-test on pathway.

Measures	human	arabidopsis	yeast
InteGO vs. InteGO2	$<$ 1.0e-07	8.1e-02	$<$ 1.0e-07
average vs. InteGO2	$<$ 1.0e-07	$<$ 1.0e-07	$<$ 1.0e-07
fake vs. InteGO2	$<$ 1.0e-07	$<$ 1.0e-07	$<$ 1.0e-07
HRSS vs. InteGO2	$<$ 1.0e-07	$<$ 1.0e-07	$<$ 1.0e-07
Resnik vs. InteGO2	$<$ 1.0e-07	$<$ 1.0e-07	$<$ 1.0e-07
Schliker vs. InteGO2	$<$ 1.0e-07	$<$ 1.0e-07	$<$ 1.0e-07
simGIC vs. InteGO2	$<$ 1.0e-07	8.3e-01	$<$ 1.0e-07
simUI vs. InteGO2	$<$ 1.0e-07	8.3e-01	$<$ 1.0e-07
TO vs. InteGO2	$<$ 1.0e-07	$<$ 1.0e-07	$<$ 1.0e-07
Wang vs. InteGO2	$<$ 1.0e-07	9.8e-01	$<$ 1.0e-07

Table S9: The 25th percentile, median and 75th percentile value of LFC score boxplot on molecular function based on human EC. InteGO2(n) means the best n input measures of InteGO2 are removed.

	25th percentile	median	75th percentile
InteGO2 (0)	4.489	5.929	6.943
InteGO2 (1)	4.467	5.76	6.903
InteGO2 (2)	4.405	5.799	6.901
InteGO2 (3)	3.017	4.907	6.011
InteGO2 (4)	1.671	4.43	5.917

Table S10: The R-squared score with polynomial model to show the correlation between semantic similarity and sequence similarity on human.

	InteGO2	InteGO	HRSS	Resnik	Schlicker	simGIC	simUI	TO	Wang
human	0.96	0.89	0.56	0.84	0.83	0.73	0.85	0.92	0.95
